# Risk of mortality among children, adolescents, and adults with autism spectrum disorder or attention deficit hyperactivity disorder and their first-degree relatives: a protocol for a systematic review and meta-analysis of observational studies

**DOI:** 10.1186/s13643-017-0581-9

**Published:** 2017-09-15

**Authors:** Ferrán Catalá-López, Brian Hutton, Matthew J. Page, Manuel Ridao, Jane A. Driver, Adolfo Alonso-Arroyo, Jaume Forés-Martos, Diego Macías Saint-Gerons, Eduard Vieta, Alfonso Valencia, Rafael Tabarés-Seisdedos

**Affiliations:** 1 Department of Medicine, University of Valencia/INCLIVA Health Research Institute and CIBERSAM, Valencia, Spain; 2 Fundación Instituto de Investigación en Servicios de Salud, Valencia, Spain; 3 Clinical Epidemiology Program, Ottawa Hospital Research Institute, Ottawa, ON Canada; 4 School of Epidemiology, Public Health and Preventative Medicine, University of Ottawa, Ottawa, ON Canada; 5 School of Public Health and Preventive Medicine, Monash University, Melbourne, VIC Australia; 6 Instituto Aragonés de Ciencias de la Salud, Red de Investigación en Servicios de Salud en Enfermedades Crónicas (REDISSEC), Zaragoza, Spain; 7 Fundación para el Fomento de la Investigación Sanitaria y Biomédica de la Comunitat Valenciana (FISABIO-Salud Pública), Valencia, Spain; 8 Geriatric Research Education and Clinical Center, VA Boston Healthcare System, Boston, MA USA; 9 Division of Aging, Department of Medicine, Brigham and Women’s Hospital, Harvard Medical School, Boston, MA USA; 10 Department of History of Science and Documentation, University of Valencia, Valencia, Spain; 11 Unidad de Información e Investigación Social y Sanitaria-UISYS, University of Valencia-Spanish National Research Council (CSIC), Valencia, Spain; 12 Division of Pharmacoepidemiology and Pharmacovigilance, Spanish Medicines and Healthcare Products Agency, Madrid, Spain; 13 Department of Health Systems and Services, Pan American Health Organization, Unit of Medicines and Health Technologies, Washington, DC USA; 14 Hospital Clínic, Universitat de Barcelona, Institut d’Investigacions Biomèdiques August Pi i Sunyer (IDIBAPS) and CIBERSAM, Barcelona, Spain; 15 Life Sciences Department, Barcelona Supercomputing Center, Barcelona, Spain; 16 Structural Biology and Biocomputing Programme, Spanish National Cancer Research Centre (CNIO), Madrid, Spain

**Keywords:** Autism spectrum disorders, Attention deficit hyperactivity disorder, Adolescent, Adult, Child, Child development, Epidemiology, Mortality, Meta-analysis, Neurodevelopmental disorders, Systematic review

## Abstract

**Background:**

Autism spectrum disorder (ASD) and attention deficit hyperactivity disorder (ADHD) are childhood onset neurodevelopmental disorders that may persist into adulthood. ASD and ADHD tend to run in families and may have a significant negative impact on the health and longevity of those with the disorder and their relatives. The aim of this study will be to analyze the risk of mortality among children, adolescents, and adults with ASD or ADHD and their first-degree relatives.

**Methods/design:**

We will conduct a systematic review and meta-analysis of observational studies. Searches of PubMed/MEDLINE, EMBASE, PsycINFO, SCOPUS, and ISI Web of Science will be used to identify epidemiological studies. Eligible studies will be observational studies reporting study-specific data for all-cause mortality or cause-specific mortality in children, adolescents, or adults with ASD or ADHD and/or their first-degree relatives. Cohort studies and case-control studies will be included. The primary outcome will be all-cause mortality. The secondary outcome will be cause-specific mortality. Two reviewers will independently screen references identified by the literature search, as well as potentially relevant full-text articles. Data will be abstracted, and study risk of bias/methodological quality will be appraised by two reviewers independently. The methodological quality of epidemiological studies will be appraised using the Newcastle-Ottawa Scale (NOS). Conflicts at all levels of screening and abstraction will be resolved through discussion. Random-effects meta-analyses of primary studies will be conducted where appropriate. Subgroup analyses for exploring statistical heterogeneity, if feasible, will include gender, age group, ethnicity, comorbidities, classification of cause of death, and relevant study characteristics.

**Discussion:**

Our study will establish the extent of the epidemiological evidence underlying the risk of mortality among children, adolescents, and adults with ASD or ADHD and their first-degree relatives. We anticipate that our findings will be of interest to patients, their families, caregivers, healthcare professionals, scientists, and policy makers. Implications for future epidemiological research will be discussed.

**Systematic review registration:**

PROSPERO CRD42017059955.

**Electronic supplementary material:**

The online version of this article (10.1186/s13643-017-0581-9) contains supplementary material, which is available to authorized users.

## Background

Autism spectrum disorder (ASD) and attention deficit hyperactivity disorder (ADHD) are childhood onset neurodevelopmental disorders that may persist into adulthood [1–3]. ASD is characterized by deficits in social communication and reciprocal interactions as well as stereotypical behavior [2], whereas ADHD is characterized by a persistent pattern of symptoms of hyperactivity, impulsiveness, and inattention [3]. Although the etiology of ASD and ADHD remains largely unknown, a complex interaction of multiple factors is thought to contribute to the development of both conditions. ASD and ADHD present a set of behavioral problems producing significant functional impairment in social, school, or work performance and in the everyday activities of patients and their families [1–6]. In 2015, global burden estimates revealed 62.2 million people with ASD (representing 10,051,515 disability-adjusted life years) and 51.1 million people with ADHD (representing 620,074 disability-adjusted life years) worldwide [7, 8]. The burden due to premature mortality is not reflected in these numbers because the potential contribution of severe mental illness to mortality from associated causes is not considered generally [8, 9]. This is due to the manner in which causes of deaths are assigned in the International Classification of Diseases (ICD) death coding system [10, 11].

Death in people with severe mental disorders has been examined in observational studies [12–14], and in recent years, evidence has been accumulated suggesting that people with mental disorders may experience a significant reduction in life expectancy, with risk of mortality increasing with the severity of the disorder [15]. A 2012 systematic review [16] evaluating two outcomes (epilepsy and mortality) claimed that people with ASD may have a higher risk of mortality than the general population. Since then, several large observational studies on the risk of mortality in ASD or ADHD have been published [17–19]. ASD and ADHD tend to run in families and may have a significant negative impact on the health and longevity both of those with the disorder and their first-degree relatives [20, 21]. To the best of our knowledge, no systematic review and meta-analysis of epidemiological studies has specifically evaluated the mortality risk and main causes of death in ASD or ADHD.

To better understand the strength of evidence, we will conduct a systematic review and meta-analysis analyzing the risk of all-cause mortality among children, adolescents, and adults with ASD or ADHD and their first-degree relatives. A further aim will be to address cause-specific mortality and to evaluate the role of bias and potential moderators in the associations between ASD or ADHD and the risk of dying.

## Methods

The proposed systematic review will be conducted and reported in accordance with the reporting guidance provided in the Preferred Reporting Items for Systematic Reviews and Meta-analyses (PRISMA) statement [22] and the Meta-analysis Of Observational Studies in Epidemiology (MOOSE) reporting guideline [23]. This systematic review protocol is registered with the International Prospective Register of Systematic Reviews (PROSPERO) database (registration number: CRD42017059955) and has been reported in accordance with the PRISMA for Protocols (PRISMA-P) statement [24, 25] (see Additional file 1 for the checklist).

### Eligibility criteria

Studies will be selected according to the following criteria: study design and participants (exposure), comparator(s) or control group, outcome(s) of interest, and setting.

#### Study design and participants of interest

Eligible studies will be observational studies reporting study-specific data for all-cause mortality or cause-specific mortality in children, adolescents, or adults with ASD or ADHD and/or their first-degree relatives. Case-control studies, prospective cohort studies with a reference comparator, and retrospective cohort studies with a reference comparator (also known as historical cohort studies) will be included. Randomized controlled trials will be unavailable for our research question. For participants (index subjects will be patients or first-degree relatives), we will use investigator-reported definitions of ASD or ADHD according to the accepted diagnostic criteria (e.g., ninth or tenth revisions of the ICD coding system or the third, fourth, or fifth edition of the Diagnostic and Statistical Manual of Mental Disorders [DSM] criteria): ASD (ICD-9: 299.0, 299.8; ICD-10: F84) and ADHD (ICD-9: 314.00, 314.01; ICD-10: F90). We will exclude studies in which ASD or ADHD is not the exposure of interest and all-cause or cause-specific mortality is not the outcome of interest. Observational studies not presenting study-specific data (e.g., relative risks, 95% confidence intervals, numbers of cases/population, observed, and expected cases) or sufficient data for an outcome measure to be calculated (see the “Outcomes” section below) will be also excluded.

#### Comparator(s) or control group

The comparator group will be based on subjects with no history of ASD or ADHD (e.g., the general population, the community, unexposed outpatient or hospital-based controls, parents or siblings of people without ASD or ADHD).

#### Outcome(s)

The primary outcome will be all-cause mortality based on valid information sources (e.g., national vital registration system, hospital records, death certificates, verbal autopsy standards). Effect measures will include the maximally adjusted effects reported as the standardized mortality ratio (SMR), the relative risk (RR), the odds ratio (OR), and the hazard ratio (HR) for the risk of mortality between ASD and ADHD compared with controls. The secondary outcome measure will be cause-specific mortality (according to the main causes of death grouped into the ICD categories of diagnoses).

#### Setting

There will be no restriction by study setting.

### Search methods

The search strategies will be designed and carried out by the research team, which will include a senior information specialist. A date restriction will not be imposed. We will search the following electronic databases, from inception, using the same search strategy with alterations as appropriate for each database: PubMed/MEDLINE, EMBASE, PsycINFO, SCOPUS, and ISI Web of Science. Search terms will include key words, controlled vocabulary and text words related to autism, attention-deficit hyperactivity, mortality, and epidemiological studies. Details of the draft search strategy for PubMed/MEDLINE are provided in an additional file (see Additional file 2). Additional studies will be identified from the reference list of articles and relevant reviews or documents. We will also attempt to identify additional studies by contacting authors of included studies or reviews. There will be no restriction by language of publication, and we will arrange for translation where necessary.

### Screening and selection procedure

Two reviewers will screen all articles identified from the search independently. First, titles and abstracts of articles returned from initial searches will be screened based on the eligibility criteria outlined above. Second, full texts will be examined in detail and screened for eligibility. Third, references of all considered articles will be hand searched to identify any relevant study missed in the search strategy. Any disagreement between reviewers will be resolved by discussion to meet a consensus. A flow chart showing details of studies included and excluded at each stage of the study selection process will be provided following the PRISMA recommendations [22].

### Data extraction

From each eligible report, two reviewers will extract information independently on first author, year of publication, neurodevelopmental disorder (ADHD or ASD), index subjects (patients, parents of patients, or siblings of patients), the general characteristics of participants (age, gender, ethnicity, social status, and comorbidities such as intellectual disability, substance abuse, epilepsy, and other medical or psychiatry conditions), epidemiological design (cohort or case-control, prospective, or retrospective), the country of study, the follow-up period, the setting (mixed, inpatient, outpatient, or community), the sample size, the outcomes assessed (including definitions and confounding factors that were taken into consideration), the number of cases and controls (in case-control studies) or the number of cases and population participants (in cohort studies), and/or the maximally adjusted effect measure (e.g., relative risk, hazard ratio, or standardized mortality ratio) with 95% confidence intervals. We will use pre-designed forms that will be piloted initially on a small number of included studies. We will also attempt to contact authors of primary publications and/or collaborators for missing outcome data or unclear information in order to maximize available data.

### Quality assessment

The methodological quality and bias of epidemiological studies will be appraised using the Newcastle-Ottawa Scale (NOS) for observational studies [26]. Using the NOS tool, each study is judged on 8 items, categorized into 3 groups: the selection of the study groups, the comparability of the groups, and the ascertainment of either the exposure or outcome of interest for case control or cohort studies, respectively. Stars are awarded for each quality item, and the highest quality studies are awarded up to 9 stars. We will consider studies with 0–3, 4–6, and 7–9 stars to represent low, moderate, and high quality, respectively. The quality for each observational study will be independently assessed by two reviewers. Discrepant scores will be resolved by discussion and consensus. Findings from quality assessment will inform supplemental analyses (see “Data synthesis” section below).

### Data synthesis

We will calculate summary effect estimates comparing the mortality in people with ADHD or ASD with the mortality in the reference control group. Statistical combination of data from two or more observational studies in a meta-analysis will be conducted and reported separately for ADHD and ASD. We will estimate the summary effect size and its 95% confidence interval using the inverse variance method based on the DerSimonian and Laird random-effects model [27]. The random-effects model is selected a priori to synthesize the epidemiological data, as it considers both within-study and between-study variation by incorporating the heterogeneity of effects into the overall analyses. If primary studies report results separately for men and women (or other subgroups), we will combine the subgroup-specific estimates using a fixed-effects model to generate an estimate for both subgroups combined so that each study was represented only once in the analyses. Patient studies with mortality data will be included in the main meta-analyses and first-degree relative studies separately in different (secondary) meta-analyses. We will evaluate statistical heterogeneity by estimating the variance between studies using the Cochran’s *Q* test [28] and *I* ^2^ statistic [29]. The Cochran’s *Q* test is obtained by the weighted sum of the squared differences of the observed effect in each study minus the fixed summary effect. The *I* ^2^ statistic is the ratio of variance between studies over the sum of the variances within and between studies and ranges between 0 and 100% (with values of 0–25% taken to indicate low heterogeneity and 75–100% to indicate considerable heterogeneity). We will also estimate the 95% prediction interval [30] where appropriate, which further accounts for between-study heterogeneity and evaluates the uncertainty for the effect that would be expected in a new observational study addressing that same association. Small study effects will be investigated by visual inspection of funnel plots (where appropriate) and formally tested using Egger’s [31] and Begg’s test [32], with the results considered to indicate potential small study effects when *P* < 0.10.

If sufficient studies are identified, we will present subgroup analyses to attempt to explain any potential observed between-study heterogeneity. The potential moderators (covariates) considered will be gender (male or female), diagnostic criteria used (e.g., DSM or ICD and their versions), age group at first diagnosis (children and adolescents [< 18 years old] versus adults [≥ 18 years old]), ethnicity (e.g., white or non-white), number of comorbidities (e.g., 0, 1, 2, or ≥3), medical or neuropsychiatric comorbidity (e.g., presence or absence of a comorbid disorder with ASD or ADHD), index subjects (patients, parents of patients, or siblings of patients), country or geographic region, cohort/sample size (< 500, 500–1000, or > 1000 participants), setting (mixed, inpatient, outpatient, or community/general population), population-based (yes or no), follow-up period (0–1, > 1–5, or > 5 years), year of publication as a proxy of changes in clinical practice over time (before 2000 or in 2000 and after), study quality (low or high-moderate quality), and effect measure adjusted for potential confounders (age, sex, or other). We will also explore the contributions from natural and unnatural causes of death. Unnatural deaths will be defined on the basis of ICD codes (ICD-10: V01-Y98 or ICD-9: E800-999); the remaining deaths will be classified as natural.

To further assess the consistency of evidence over time, we will conduct cumulative meta-analyses in the order of publication year [33, 34].

Finally, we will apply a set of criteria to conclude whether the evidence may be considered convincing, probable, limited-suggestive, limited-not conclusive, or unlikely. For this, we will follow the Global Burden of Disease Study approach for risk factors [35–37] which is also being applied in ongoing evidence syntheses of central nervous system disorders and cancer [38, 39]. “Convincing evidence” consists of biologically plausible associations between exposure and outcome established from multiple epidemiological studies in different populations. Evidentiary studies must be substantial, include prospective observational studies and, where relevant, epidemiological studies of sufficient size, duration, and quality, and show consistent effects. A convincing relationship should be robust enough to be highly unlikely to be modified in the foreseeable future as new evidence accumulates. “Probable evidence” is similarly based on epidemiological studies with consistent associations between exposure and outcome but shortcomings in the evidence exist, such as insufficient trials or prospective observational studies available. “Limited-suggestive evidence” represents too limited evidence to permit a probable or convincing causal judgment, but where there is evidence suggestive of a direction of effect. “Limited-not conclusive evidence” consists of information that is so limited that no firm conclusion can be made for a number of reasons (e.g., the evidence might be limited by the amount of evidence in terms of the number of studies available, by inconsistency of direction of effect, by poor quality of studies, or by any combination of these factors). “Substantial effect on risk unlikely” consists of evidence strong enough to support a judgment that a particular exposure is unlikely to have a substantial causal relation to a cancer outcome. The evidence should be robust enough to be unlikely to be modified in the foreseeable future as new evidence accumulates [38, 39].

We will also use the Grading of Recommendations Assessment, Development, and Evaluation (GRADE) methodology for evaluating the quality of evidence [30–42] as follows: high-quality evidence (further research is very unlikely to change our confidence in the estimate of effect), moderate-quality evidence (further research is likely to have an important impact on our confidence in the estimate of effect and may change the estimate), low-quality evidence (further research is very likely to have an important impact on our confidence in the estimate of effect and is likely to change the estimate), or very low-quality evidence (very uncertain about the estimate of effect).

### Software

All analyses will be conducted in Stata version 13 or higher (StataCorp LP, College Station, Texas, USA), using the metan (for fixed- and random-effects meta-analysis), metacum (for cumulative meta-analysis), and metabias and metafunnel (for small study effects analysis) [43].

## Discussion

This protocol describes a systematic review and meta-analysis of observational studies evaluating the risk of mortality among children, adolescents, and adults with ASD or ADHD and their first-degree relatives. We are not aware of another systematic review and meta-analysis addressing this specific issue.

This systematic review will summarize the methods used and results of epidemiological studies and will establish the extent of the accumulated body of evidence underlying the research question, in a rigorous and replicable way. A key challenge is that based on knowledge from previous reviews in neurodevelopmental disorders, we anticipate identifying studies using different study designs, diverse durations, small sample sizes, and variable quality of reporting methods and results. Further, the possibility of reporting bias (mainly, for unpublished data on cause-specific mortality) could be a potential limitation of this study.

Any amendments made to this protocol when conducting the review will be outlined and reported in the final paper. This review will also identify gaps in knowledge; thus, implications for future epidemiological research will be discussed in the final paper. We anticipate that our findings will be of interest to patients with ASD or ADHD, their families and caregivers, clinicians and other healthcare professionals, scientists, and policy makers. No ethical approval will be required for the performance of this systematic review and meta-analysis.

## Additional files

Additional file 1: PRISMA-P checklist. (DOCX 24 kb)

Additional file 2: Key terms for PubMed/MEDLINE search. (DOCX 20 kb)

## Abbreviations

ADHD: Attention deficit hyperactivity disorder
ASD: Autism spectrum disorder
DSM: Diagnostic and Statistical Manual of Mental Disorders
GRADE: Grading of Recommendations Assessment, Development, and Evaluation
ICD: International Classification of Diseases
NOS: Newcastle-Ottawa Scale
PRISMA-P: Preferred Reporting Items for Systematic Reviews and Meta-Analyses extension for Protocols
