# Supplementary material for: Risk of mortality among children, adolescents, and adults with autism spectrum disorder or attention deficit hyperactivity disorder and their first-degree relatives: a protocol for a systematic review and meta-analysis of observational studies
Source: Syst Rev. 2017 Sep 15;6:189. doi: 10.1186/s13643-017-0581-9 (PMC5603059; doi:10.1186/s13643-017-0581-9)
Supplement: Supplementary file 2 — Key terms for PubMed/MEDLINE search. (DOCX 20 kb) [file 13643_2017_581_MOESM2_ESM.docx]

**Additional file 2**

**Key terms for PubMed/MEDLINE search.**

| **Search** | **Query** |
| --- | --- |
| #1 | **“(autism spectrum disorder* OR autism OR autistic OR pervasive developmental disorder* OR Asperger OR attention deficit disorder with hyperactivity OR attention deficit/hyperactivity disorder OR adhd OR hyperkinetic* OR inattent* OR impulsivity OR hyperkinesis OR tdah)”** |
| #2 | **“(epidemiology OR epidemiologic* OR cohort stud* OR longitudinal stud* OR case-control stud*)”** |
| #3 | **“(mortality OR death* OR survival* OR fatal*)”** |
| #4 | **#1 AND #2 AND #3**  **No limits** |
